# Using Local Ecological Knowledge to Search for Non-Native Species in Natura 2000 Sites in the Central Mediterranean Sea: An Approach to Identify New Arrivals and Hotspot Areas

**DOI:** 10.3390/biology12091158

**Published:** 2023-08-23

**Authors:** Patrizia Perzia, Tiziana Cillari, Giuseppe Crociata, Alan Deidun, Manuela Falautano, Giulio Franzitta, Johann Galdies, Teresa Maggio, Pietro Vivona, Luca Castriota

**Affiliations:** 1 Unit for Conservation Management and Sustainable Use of Fish and Marine Resources, Department for the Monitoring and Protection of the Environment and for the Conservation of Biodiversity, Italian Institute for Environmental Protection and Research, Lungomare Cristoforo Colombo 4521 (Ex Complesso Roosevelt), Località Addaura, 90149 Palermo, Italy; 2 Oceanography Malta Research Group (OMRG), Department of Geosciences, Faculty of Science, University of Malta Tal-Qroqq Campus, MSD 2080 Msida, Malta; 3 Department of Research Infrastructure for Marine Biological Resources, Stazione Zoologica Anton Dohrn, Via Giardini Molosiglio, 80133 Naples, Italy

**Keywords:** *Caulerpa* spp., CIMPAL, citizen science, cumulative impact, Local Ecological Knowledge, marine nonindigenous species, *Siganus* spp.

## Abstract

**Simple Summary:**

Biological invasions are one of the most urgent issues to be managed in order to avoid the risk of endemic biodiversity loss. Among management strategies, the monitoring of non-native species is needed to make appropriate decisions. To complement the standard monitoring, citizen science is increasingly being used. Within citizen science, the approach of Local Ecological Knowledge (LEK) proved to be useful in the monitoring of non-native species. A LEK survey was carried out in 10 Sicilian and Maltese Natura 2000 sites and was addressed to local fishers and SCUBA divers in order to help in the early detection of non-native species. The occurrence of 24 selected marine non-native species was investigated through the use of a questionnaire for the LEK survey. Potential hotspot areas of invasion were identified by using six indicators: the occurrence of newly introduced nonindigenous species, the cumulative impacts of invasive alien species (CIMPAL), and the relative importance of species on the cumulative impacts (D1, D2, D3, and D4). The respondents confirmed the presence of 22 species since 2000 and reported 10 new ones in the investigated areas. The highest CIMPAL values were observed in Sicily in the Fondali dell’isola di Capo Passero and in the MPA Isole Pelagie and the lowest on the western coast of Malta (MT0000101, MT0000102, MT0000103, and MT0000104). The four top-priority species according to indicators D1–D4 were the algae *Caulerpa cylindracea* and *C. taxifolia* and the fishes *Siganus luridus* and *S. rivulatus*.

**Abstract:**

The management of biological invasions is among the most urgent of global challenges and requires a significant monitoring effort to obtain the information needed to take the appropriate decisions. To complement standard monitoring, citizen science is increasingly being used. Within citizen science, the approach of collecting and investigating Local Ecological Knowledge (LEK) proved to be useful in the monitoring of non-native species. A LEK survey was carried out in 10 Sicilian and Maltese Natura 2000 sites in order to help in the early detection of non-native species. The survey was addressed to local fishers and SCUBA divers in order to investigate the occurrence of 24 selected marine non-native species and to identify potential hotspot areas of invasion through the use of six indicators: the occurrence of newly introduced nonindigenous species, the cumulative impacts of invasive alien species (CIMPAL) and the relative importance of species on the cumulative impacts (D1, D2, D3, and D4). The respondents confirmed the presence of 22 species since the year 2000 and reported 10 new ones registered in the investigated areas. The highest CIMPAL value was observed in two Sicilian Natura 2000 sites (ITA090028 and ITA040014) and the lowest on the western coast of Malta (MT0000101, MT0000102, MT0000103, and MT0000104) The four top-priority species according to indicators D1–D4 were *Caulerpa cylindracea*, *C. taxifolia*, *Siganus luridus* and *S. rivulatus*. The study produced a valid and useful scientific output to suggest and address management strategies to monitor the establishment of the non-native species.

## 1. Introduction

The management of biological invasions is amongst the most urgent of global challenges, and particularly in environments of high connectivity, such as marine waters where usually there are less physical barriers to impede organisms from dispersing to new areas when compared to terrestrial or freshwater environments. Management-related problems are even more complex when the jurisdiction of the marine area/s in question happen to be divided amongst more than one nation. Although exotic marine species generally suffer extremely high levels of post-colonisation mortality, resulting in a slowdown or even a failure of the establishment process from taking place in the new area [1,2], some of them do succeed due to intrinsic invasiveness features they might possess (such as high fecundity or fast growth and high plasticity/tolerance to wide a range of conditions) or because they happen to find particularly favourable conditions for establishment in the new environment. In the latter case, invaders can significantly impact indigenous species, habitats, ecosystems and/or ecosystem services [3], although in most cases awareness of these impacts remains limited [4,5,6]. Most new colonisations are detected only after a certain period of time has elapsed, a period of time corresponding to distinct lag phases typical of biological invasions during which the invasive species would not be showing any signs of evident population growth [7,8]. When the species’s presence in the new environment is finally recorded, it is generally too late or costly to implement any effective containment and control operations to help mitigate or manage the said invasion [9].

Currently, European and Mediterranean Basin directives, including the Marine Strategy Framework Directive (MSFD) and the Barcelona Convention through the Ecosystem Approach (EcAp), promote environmental monitoring for the detection of the newly arrived nonindigenous species (NIS—the term NIS is used to indicate species introduced in a new environment through direct or indirect human intervention, according to [10])) in different areas. However, the investment needed to comply with these directives, both in terms of monetary resources and human resources, does not guarantee a full coverage of marine spaces, and thus many introductions can elude these monitoring efforts. For this reason, any proposed monitoring strategy ought to include more than one detection method. The scientific community is now recognising the importance of actively involving citizens in the observation of visible natural phenomena in order to use any such knowledge for environmental management purposes, such as participation in monitoring activities (and thus serve as early warning systems), or the use of specific participatory tools used according to guidelines provided by researchers (i.e. the citizen science approach) [11,12,13]. One of the key strengths of the citizen science approach is the ability to cover larger geographical areas at a significantly lower cost when compared to scientific surveys. Thus, a wide network of citizen scientists can reduce the time taken to first detect a NIS and can thus be utilised to track the spread of said NIS [14] giving environmental managers the ability to intervene earlier with any mitigation measures.

**Figure 1:**
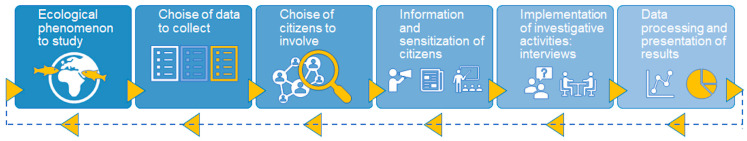
Local Ecological Knowledge (LEK) flow chart.

Within citizen science, the acquisition of Local Ecological Knowledge (LEK) should be applied according to a specific research methodology involving the collection of evidence for, and observations on, the ecological phenomena of interest [15]. LEK surveys depend on citizen experience in the specific field/s of interest as well as on adequate training delivered to the interviewers by the trainer to ensued that they communicate effectively with the interviewees. In order to obtain reliable data through LEK-based protocols, appropriate strategies should be adopted e.g.,: (i) the intermediation by accredited key informants to effectively persuade citizens to cooperate; (ii) organising meetings in formal places (such as sites of marine protected areas, port authorities, naval leagues, etc.) and/or in informal ones (port areas, diving centres, etc.) to inform and sensitise the citizens involved and to encourage them to share their experiences; and (iii) the creation of a network for future collaboration/s. Once the interviews have been carried out and all the data have been collected, they must be validated and subsequently processed. It is important that the results of the study are shared and disseminated as widely as possible in order to deepen and consolidate the knowledge acquired by citizen scientists and to ensure the long-term sustainability of these data acquisition and awareness-generation activities (Figure 1).

Several studies have shown that data collection via LEK can be useful to investigate any trends in the occurrence of marine species as well as in evaluating the presence of NIS in an area as a complementary tool to standard monitoring (i.e., organism collection and/or visual census and subsequent identification), supporting environmental management and decision making [16,17,18,19,20]. Furthermore, LEK activities allow the investigator/s to retrieve unpublished documentation (photos, videos, and/or samples), which improve the knowledge on the distribution or invasion history of the species being investigated. 

The integration of the acquired data with previously published data may help in the identification of eventual hotspot areas, especially for species that are not detectable through standard methods and whose information is incomplete in the literature. 

**Figure 2:**
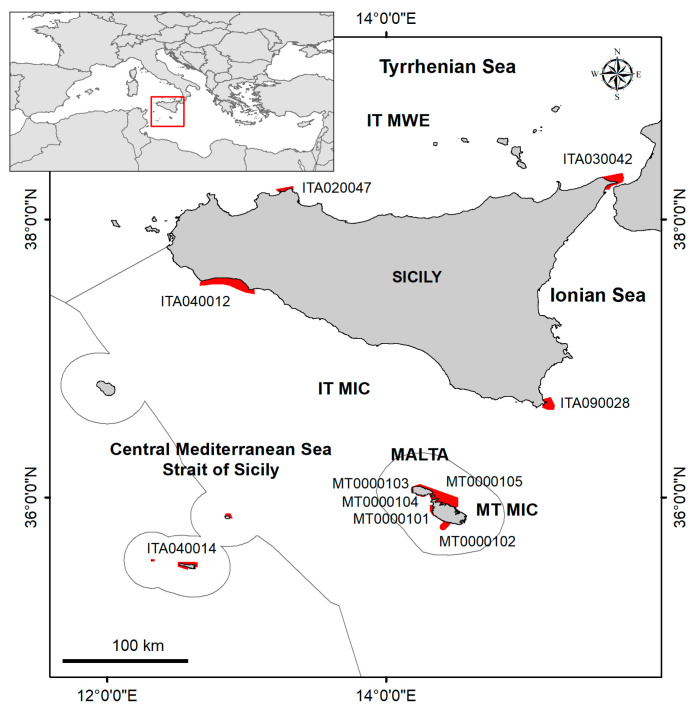
Sicilian and Maltese Natura 2000 sites areas (in red) investigated through the LEK survey. The grey lines delimit the Italian (IT) and Maltese (MT) marine subregions, according to the Marine Strategy Framework Directive (Committee in November 2016). MWE = Western Mediterranean Sea; MIC = Ionian Sea and the Central Mediterranean Sea.

Within the framework of the project HARMONY (Interreg V-A Italia Malta, 2014–2020), a LEK survey was carried out at 10 Sicilian and Maltese N2K sites (Figure 2) on aspects related to early species detection.

The HARMONY project aimed to validate a set of monitoring and control measures, including LEK, in two cross-border countries: Italy and Malta. The cross-border nature of Italy and Malta, with both countries depending on shared biological resources and similar social and economic structures, urged the setup of a transboundary observatory within the strait of Sicily (the HARMONY project) to develop common Early Detection & Rapid Response (EDRR) tools and produce a common strategy for NIS management. The LEK survey carried out as part of this project in question was addressed to citizens living in close contact with the marine environment, i.e., local fishers and SCUBA divers, in order to investigate the introduction and occurrence of selected marine non-native (In this paper the term nonnative is used for species not previously occurring in the area, including those introduced through human activities (NIS), those introduced as a consequence of climatic changes; those of unknown or doubtful origin are usually indicated as cryptogenic) species. The acquired data were used to identify potential hotspot areas for invasion by non-native species through the use of indicators, which in turn were used to suggest and address management strategies.

## 2. Materials and Methods

### 2.1. Study Area

The Strait of Sicily (or Sicily Channel) is located in the Central Mediterranean Sea, between southern Sicily and the North African coast (Tunisian peninsula), 140 km wide. Owing to its geomorphological, oceanographic and climatic conditions, this area supports a unique and exclusive ecosystem with a rich biodiversity [21]. From a biogeographical point of view, it is a well-defined area that connects the western and eastern halves of the Mediterranean Sea [21], presenting a bridge for species originating from the Atlantic Ocean or the Red Sea to spread into the eastern and western halves of the Mediterranean respectively [22].

The LEK activity was carried out at ten different Natura 2000 (N2K) sites (EU Habitats Directive), five in Italy (Sicily) and five in Malta (Figure 2), including eight Special Areas of Conservations (SACs), one Special Protection Area (SPA), and one proposed SCI (pSCI) (Table 1). Seven of these are also Marine Protected Areas (MPAs) (While Natura 2000 sites are specifically designated to protect areas of critical importance at the EU level for a number of species/habitats listed in the Habitats and Birds Directives, MPAs are established under national or regional laws for a variety of different purposes and may also cover different species/habitats than those in the Natura 2000 network).

The sites were selected according to their distribution in the entire study area of the HARMONY Project.

All the selected sites are fully marine, with the exception of ITA030042, whose marine component amounts to 29% of its total area.

The habitat types (according to the terminology used in Annex I of EU Habitat Directive) characterising these sites are:

- Sandbanks, which are slightly covered by sea water all the time (Habitat Code: 1110)
- *Posidonia* beds (Posidonion oceanicae) (Habitat Code: 1120)
- Coastal lagoons (Habitat Code: 1150)
- Reefs (Habitat Code: 1170)
- Submerged or partially submerged sea caves (Habitat Code: 8330).

‘Posidonia beds’ and ‘Coastal lagoons’ are listed as priority habitat types within this Directive.

The rationale for selecting these particular habitats was based on the information included in the Natura 2000 standard data forms [23,24,25,26,27,28,29,30,31,32], cross-checked with the EUNIS 2019 classification of the broad-scale seabed habitat map for Europe [33], and using the crosswalks between the EUNIS marine habitats classification and the one based on Annex I Habitats (reported in the excel file ‘EUNIS marine habitats classification 2022 with crosswalks to Annex I in separate rows’ in [34].

### 2.2. Data Collection

The LEK surveys were carried out in 2019, through semi-structured individual face-to-face interviews using a questionnaire (modified from Garrabou et al. [35]) designed to investigate the occurrence/incidence of non-native species. The questionnaire targeted citizens that carry out their professional and/or leisure activities in the marine environment, namely professional and recreational fishers and SCUBA divers. 

Twenty-four non-native taxa (Table 2) were selected according to one or more of these defined criteria: (1) recorded or expected to arrive soon in at least one of the two countries; (2) degree of invasiveness; (3) easily identifiable morphological characters; (4) recruitment from local fishing systems and/or detection in coastal areas by *visual census*. Information on the occurrence of these non-native species in the Siculo-Maltese area and neighbouring areas was obtained from the national baseline inventories of the European Union’s MSFD of 2012 [36] and subsequent updates and from literature. Two fish taxa were considered at genus level only, due to the difficulty encountered in their morphological identification at species level by nonexperts. 

Among the species of interest, we also considered the venomous fish *Plotosus lineatus*, an invasive species established along the Levantine coastline as far west as Turkey [86] and also recorded in Tunisia [87] for the hazard it poses to human health as well as for its suspected ability to displace native fish species through competition [88]. *P. lineatus* has been listed as an invasive alien species of Union concern since 2019 [89,90]. A poster with photos of the non-native species under consideration was shown to the interviewees (Supplementary File), together with other photos of morphologically similar indigenous and non-native species, by means of an electronic tablet, highlighting the main distinctive features, in order to facilitate their identification.

The following data were collected through the questionnaire:

- data on the interviewee: category (recreational fisher, professional fisher, SCUBA diver); age class; years of experience at sea (starting date of activity); fishing gear/s;
- data on the species: information about the first and subsequent sightings (date/season, site, depth, substrate type and abundance), fishing gear or other sighting method, any available documentation (photo or video);
- data on other species: furthermore, interviewees were also asked to report any other species never captured/seen before.

Some strategies were adopted in order to obtain more reliable data/information on the species investigated through the LEK survey (Table 3).

Citizen observations were discarded if: (i) the observation was not conclusive or was not comprehensively described; (ii) the species was suspected to have been misidentified with other similar species; (iii) the respondent answered reluctantly, demonstrating reticence and/or non-cooperation; (iv) the respondent showed excessive confidence through a know-it-all attitude, which dispelled any possible dissenting opinions. In case of an uncertain observation date, the year was indicated as ≤2019. The validated observations were then collected in a database for data management and analyses.

In order to keep the LEK network active, the respondents were asked to keep reporting and documenting future sightings.

### 2.3. Data Management and Analyses

#### 2.3.1. Interviewees and Species Data Analysis 

The data obtained through the questionnaires were organised in a database which was subsequently validated according to the abovementioned criteria and analysed. The citizen categories were characterised in terms of age, experience and activity. The distribution of the number of interviews as well as the citizen categories for each site were computed. The number of non-native species records and the number of non-native species per N2K site and neighbouring areas (considering a buffer of 4 km) were also mapped. The data obtained from LEK and the post processing were mapped using ArcMAP PRO 10.3.

#### 2.3.2. Impact of LEK on the Primary Criterion D2C1 of MSFD

MSFD considers NIS among the descriptors of good environmental status (GES) for marine waters, namely through Descriptor 2 (D2) i.e., “Non-indigenous species introduced by human activities are at levels that do not adversely alter the ecosystems”. As a primary criterion (D2C1) to determine GES, the minimisation or even better the zeroing of the number of nonindigenous species newly introduced via human activity into the wild over a six-year assessment period was adopted [91]. In this context, in order to evaluate the usefulness of LEK as a complementary monitoring approach, LEK records were used to assess the presence of new introductions for each national subregion compared to the most recent baseline inventory updated up to 2012 [36] and subsequently updated from the literature up to the current LEK activity year (i.e., 2019). 

#### 2.3.3. Hotspot Areas of Non-Native Species’ High Impact by LEK Data

In order to identify the main hotspot areas for the impact of non-native species, the LEK data were used to calculate the Cumulative IMPacts of invasive ALien species (CIMPAL) indicator on marine ecosystems developed by Katsanevakis et al. [5] and modified for this study. 

For each N2K site, the cumulative impact scores of the present non-native species on a site, I_c_, were estimated as:

$$I_{c}=\sum_{i=1}^{n}\sum_{j=1}^{m}A_{i}H_{j}w_{ij},$$

where

$A_{i}$ = Index of the state of the invasive alien species population i in the specific N2K site. We used the presence/absence (1/0) data for this state variable.
$H_{j}$ = Index of the extent of habitat j in a specific N2K site. We used the habitat presence/absence (1/0) data for this state variable (Table 1).
$w_{i,j}$ = Impact weight for species i on habitat j present in the specific N2K site (Table 4).
n, m = The numbers of invasive alien species and marine habitats, respectively, that were included in the analysis.

The impact weight for species i and habitat j were defined according to the classification proposed by Katsanevakis et al. [5], and w_i,j_ was assumed to be spatially constant in the whole N2K site. No impact values were reported for species not inhabiting the corresponding habitats (Table 4). 

The relative importance of species on the cumulative impacts (D1–D4) [5] across the N2K sites was also investigated as:

- The total area of occurrences per species as the total number of N2K sites (D1);
- The number of N2K sites with an impact weight score >0 per species (D2);
- The sum of the impact weight score of the species across all N2K sites (D3);
- The average impact weight across the range of occurrence (i.e., estimated across the number of N2K sites where the species was present) (D4).

The CIMPAL and D1–D4 indicators were calculated considering the non-native species (n) found within the N2K sites and in the neighbouring area and five habitat types (m) (Table 1).

## 3. Results

### 3.1. Data on the Interviewees and Species

In total, 127 citizens were interviewed, 113 in the Sicilian areas and 14 in the Maltese areas. The sample of respondents was composed almost entirely of males (93.7%) and the female respondents were exclusively SCUBA divers.

**Figure 3:**
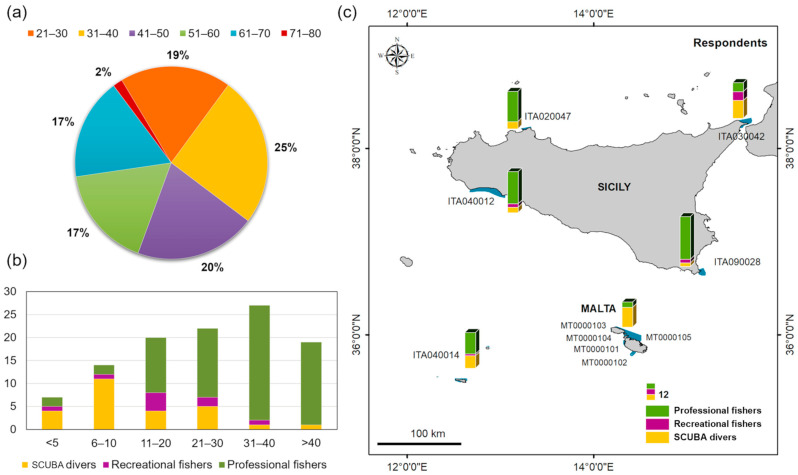
Respondents’ sample characterization: (**a**) age class group; (**b**) years of experience at sea; (**c**) distribution per N2K site. The blue polygons in the map indicate the Sicilian and Maltese Natura 2000 sites investigated.

The sample was almost uniformly distributed across all age groups (Figure 3a), with the exception of the 71–80 age group which only had two respondents. The most represented age class was 31–40 (25.0%). About 62% of respondents had more than 20 years of experience (Figure 3b).

The majority of respondents were professional fishers (60.6%), followed by SCUBA divers (31.5%) and recreational fishers (7.9%). Gillnets were the most commonly used gear among professional fishers (71%), followed by longlines (13%), trawl (10%), traps (5%) and purse seine (1%).

**Figure 4:**
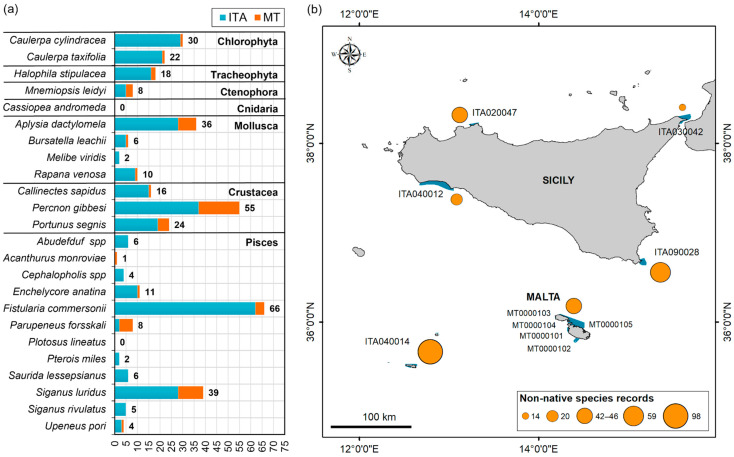
(**a**) Number of records per species in Sicily and Malta. (**b**) Number of non-native species records per N2K sites and neighbouring areas (buffer of 4 km); class ranges are shown using the features value. The blue polygons in the map indicate the Sicilian and Maltese Natura 2000 sites investigated.

Figure 4a shows the number of records per species obtained during the LEK survey, distinguished between Italy and Malta. The occurrences covered a period ranging from 1990 to 2019. The respondents reported the presence of 22 out of 24 non-native species, *Plotosus lineatus* and *Cassiopea andromeda* were the species not recorded during the interviews in the study area. Of the 401 records of non-native species reported by the respondents, 312 in Sicily and 67 in Malta were considered reliable, for a total of 379 records belonging to 22 species.

Two hundred and seventy-nine occurrences were recorded within the N2K sites and in the neighbouring areas (buffer of 4 km), with 100 in other areas. In the Sicilian area, *Fistularia commersonii* was by far the most reported NIS (55 records), especially in site ITA040014 (29 records). *Percnon gibbesi* (32 records) and *Siganus luridus* (34 records) were the two other most reported species. In the Maltese area, the two most reported NIS were *Percnon gibbesi* (18 records) and *Siganus luridus* (11 records). Figure 4b shows the number of non-native species records per N2K sites and neighbouring areas. In the Sicilian areas, 233 records of non-native species were detected within the N2K sites and neighbouring areas, with 79 outside. The highest numbers were recorded in the ITA040014 Fondali delle Isole Pelagie site (98 records) and in the ITA090028 Fondali dell’Isola di Capo Passero (59 records). 

In the Maltese areas, 67 records of non-native species were detected of which 21 were outside the N2K sites and neighbouring areas (Figure 4b). The highest number was recorded in MT0000102 Żona fil-Baħar fl-Inħawi ta’ Għar Lapsi u ta’ Filfla (17 records) and MT0000105 Żona fil-Baħar fil-Grigal ta’ Malta (16 records).

The species *A. dactylomela*, *C. sapidus*, *C. cylindracea*, *C. taxifolia*, *F. commersonii*, *H. stipulacea*, *P. gibbesi*, *P. segnis*, *R. venosa*, *S. lessepsianus* and *S. luridus* were all sighted more than once by the same observer.

**Figure 5:**
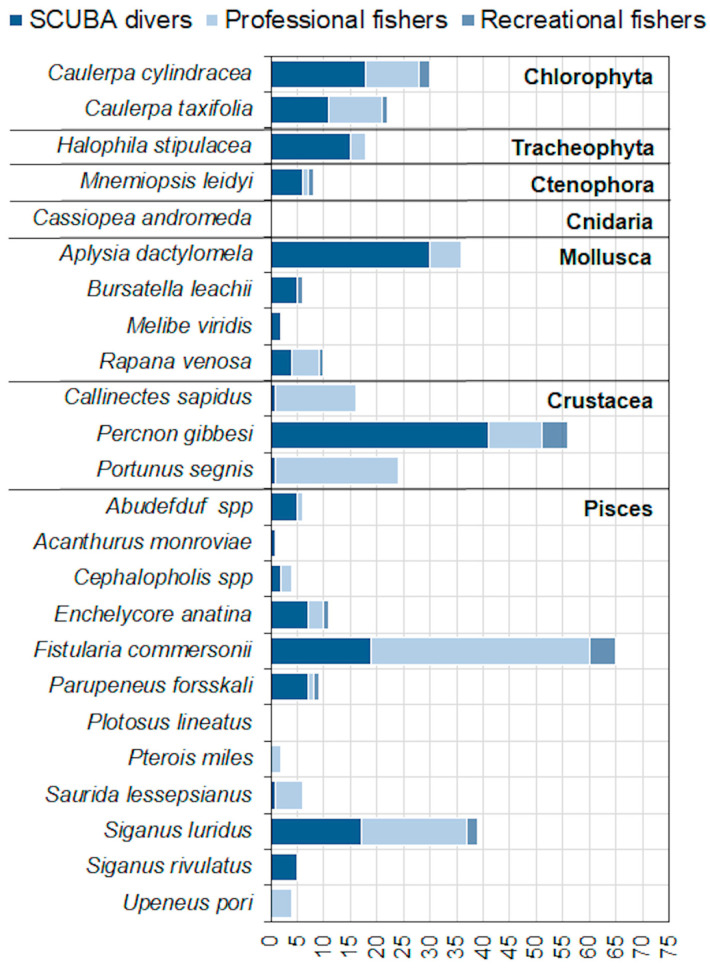
Frequency of species sighting by the three categories of citizens interviewed.

Most species were reported by all three categories of citizens interviewed. The highest number of records was reported by the SCUBA diver category (198 records), followed by professional (161 records) and recreational (20) fishers. Professional fishers generally reported more fish and blue crabs (*C. sapidus* and *P. segnis*) while SCUBA divers reported more the other categories of species (Figure 5).

### 3.2. D2C1, CIMPAL and D1–D4 Indicators

Out of 24 species investigated, respondents confirmed the presence of 22 species since 2000 and reported new ones not yet registered in the MSFD subregions (primary criterion D2C1): 11 species already known in the IT MWE subregion plus 4 new, 19 species in the IT MIC subregion plus 2 new, 12 species in the MT MIC subregion plus 4 new (Table 5). The records of *A. dactylomela*, *B. leachii*, *F. commersonii*, *P. gibbesi* and *P. segnis* were validated by documentary material (photo/video). *A. dactylomela* in IT MWE and IT MIC and *E. anatina* in IT MIC were reported before the date reported in the literature. 

**Figure 6:**
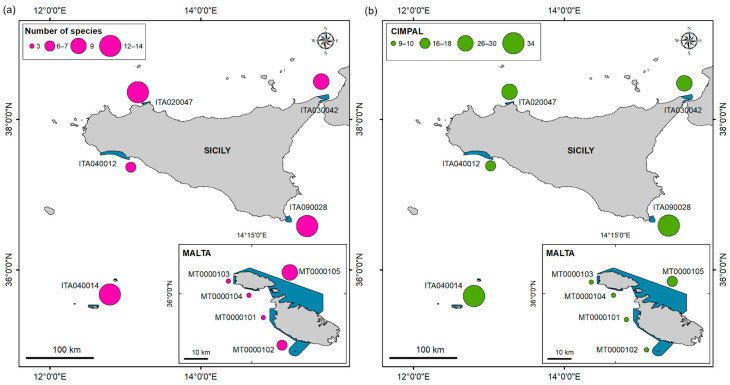
(**a**) Number of non-native species per N2K sites and neighbouring areas (considering a buffer of 4 km); (**b**) results of the sum of the Cumulative IMPacts of invasive ALien (CIMPAL) for the 22 non-native species on five habitats. Class ranges are shown using features value. The blue polygons in the maps indicate the Sicilian and Maltese Natura 2000 sites investigated.

The numbers of non-native species per N2K sites and neighbouring areas are shown in Figure 6a. Three sites that were all in Sicily had the highest number of species (12–14); four sites had a medium to high value ranging 6–7 to 9, and three sites had a low value (3). In general, the highest numbers of non-native species were observed in Sicilian N2K sites and the lowest on the western coast of Malta.

The CIMPAL scores are shown in Figure 6b. The indicator showed spatial heterogeneity in the study areas and was able to differentiate between N2K sites; it was possible to identify areas of stronger impact (CIMPAL = 34) and areas of lesser impact (CIMPAL = 9). Two sites had the highest impact scores, in southern Sicily and in the Pelagian Islands (ITA090028 and ITA040014), four sites had a medium to high value ranging from 16 to 30, and four sites that had a low value of 9–10. In general, the highest impact scores were observed in Sicily N2K sites and the lowest on the western coast of Malta.

**Figure 7:**
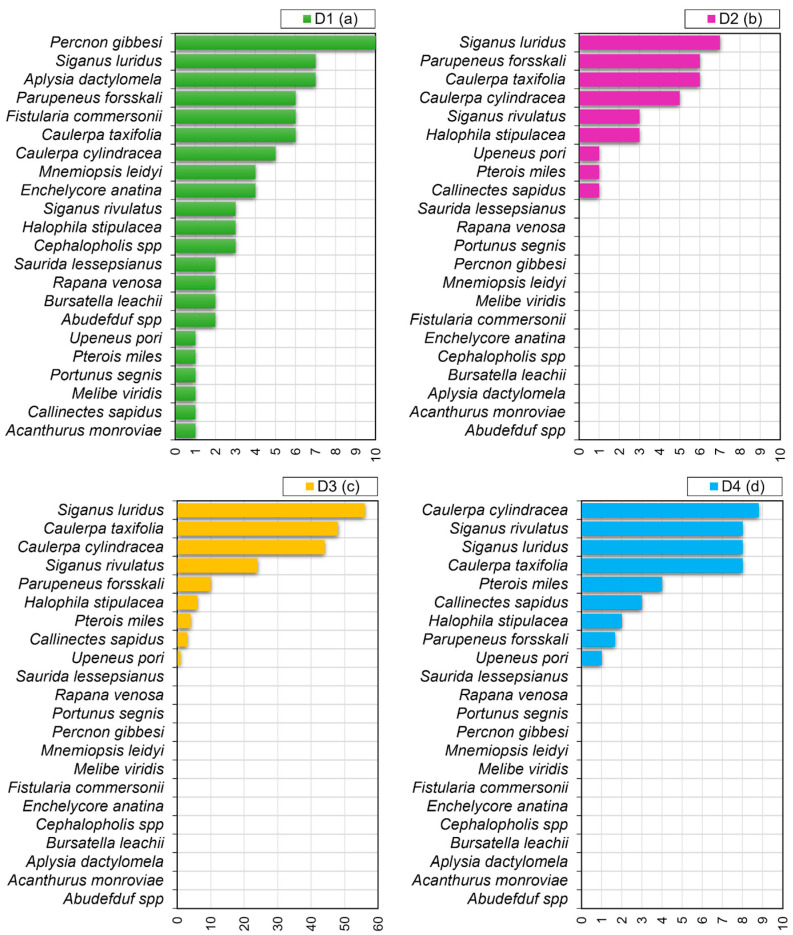
Relative importance of the 22 invasive non-native species as assessed by the indicators D1–D4. (**a**) The results of indicator D1 that reflects the total area of occurrences as a number of N2K sites; (**b**–**d**) The results of indicators D2–D4 showing the nine non-native species that scored the highest: D2 as the number of N2K sites per species with impact > 0; D3 as the sum of impact score of the species across the entire study areas; D4 as the average impact across the number of N2K sites.

The inventory and ranking of the most impacting species are displayed in Figure 7. The D1 indicator reflects the total number of N2K sites invaded, and at least one species was present in all ten sites. D2–D4 show the species that scored the highest in terms of impacts: *S. luridus*, *P. forsskali*, *C. taxifolia*, *C. cylindracea*, *S. rivulatus*, *H. stipulacea*, *U. pori*, *P. miles* and *C. sapidus*. D2 shows the nine species with impact > 0 and the number of N2K sites where each species was present; D3 and D4 accounts for the magnitude of impact and the importance of these species in the invaded N2K sites, respectively. 

Thirteen frequently reported species did not show relevant impacts on the habitats present in the investigated sites. The four top-priority species were the Indopacific macrophytes *C. cylindracea and C. taxifolia* and the Lessepsian fish *S. luridus* and *S. rivulatus* that had a decidedly higher impact compared to others. Other five high-priority species included *C. sapidus*, *H. stipulacea*, *P. forsskali*, *P. miles* and *U. pori*. 

## 4. Discussion

There is increasing global interest in marine alien species, especially invasive ones that may have significant and sometimes unpredictable and unmanageable impacts on the environment and on ecosystem services [4,6,92]. One of the reasons for this growing interest is to fill the current knowledge gap, regarding the inability of scientists and competent authorities to detect early-on the occurrence of new species, whether alien or range-expanding, in an area. Many initiatives are currently being undertaken under European and Mediterranean Basin directives to monitor new NIS introductions—the most recent estimates counted 874 NIS in December 2020 in European Union national marine waters [93]—but it is not always possible to detect new invasions because species can remain unnoticed for a long time until they become abundant or start causing damage. Well-established coordination and data sharing between contiguous countries such as Italy and Malta are also needed in order to detect and manage the possible spread of non-native species from one country to another [22]. Having a well-structured network and common management plan between neighbouring countries would allow early and efficient NIS detection with a subsequent rapid response, such as strengthening monitoring and surveillance actions or launching information and awareness campaigns in order to be able to intercept the first stages of the invasion, when mitigating bioinvasion is still possible [9]. 

In our specific case, the establishment of a transnational observatory in the Strait of Sicily would allow better monitoring and management of bioinvasions between Tunisia, Italy and Malta. Previous experience has shown that the invasion of one species in one of these countries also subsequently occurs in the other countries, as was the case for e.g., *Lagocephalus sceleratus* [94] and *Portunus segnis* [95].

From our experience, the implementation of the current LEK activity supports monitoring efforts in the area by tracing an overview on the most frequent occurrences of non-native coastal species in the investigated area and by confirming the preponderance of some nonindigenous species, including *Fistularia commersonii*, *Percnon gibbesi*, *Siganus luridus,* and *Aplysia dactylomela*, already known from the area and promptly recognized by respondents. Furthermore, outcomes of this current study suggest new introductions of species not previously recorded in the investigated MSFD subregions, thus affecting the primary criterion D2C1 of the MSFD, which would then be greater than that detected by the respective national monitoring programmes in both countries. Compared to the baseline updated in 2017 [36] and the subsequent updates from the literature, a total of 10 new non-native species were detected, six in Italy and four in Malta, seven of which were alien *sensu* MSFD criteria. One of these species, the crab *Callinectes sapidus*, was then recorded in Malta about two years after [96] the interviews in one of the locations indicated by the respondents (4 km from the southeast end of the site MT0000105), confirming the results of the LEK activity on this species. The occurrence of the fish *Upeneus pori* caught in 2018 near the site MT0000102 as reported by a Maltese fisher, was confirmed three years later [97]. Similarly, the fangtooth moray *Enchelycore anatina* was filmed by a SCUBA diver in July 2021 at Lampedusa (first documented record posted on the Facebook group Oddfish), right where it was reported by some respondents north of Lampedusa in 2017 (site ITA040014). However, other new introductions detected through the current LEK activity remain unconfirmed due to a lack of documentation, highlighting the importance of building and maintaining an active network of experienced detectors/informers supporting scientists in the data acquisition and validation stages. In this regard, LEK can present red flags on the yet-to-validate species that should encourage managers to focus search efforts on the species of interest in the sighting zones indicated by respondents.

According to the impact indicators used in this study, almost all the species on which attention was focused were reported by the respondents at least once in the N2K sites of the Sicilian–Maltese area (D1); the only exceptions concerned the fish *Plotosus lineatus*, which does not seem to have dispersed further from the Tunisian area where it has been reported more recently, and the jellyfish *Cassiopea andromeda,* which by virtue of its occurrence within shallow waters and lagoon habitats is not regularly encountered by fishers or by SCUBA divers. 

Of the 22 reported species, nine potentially solicit management attention due to their potential impacts on sensitive habitats (D2); however, the analysis suggests that the most urgent of these are the two rabbitfish *S. luridus* and *S. rivulatus* and the two *Caulerpa* species, *C. taxifolia* and *C. cylindracea* among these nine species (D3, D4). Rabbitfish are tropical invasive herbivores that, throughout their expansion in the eastern Mediterranean basin, have overgrazed canopy-forming algae like *Cystoseira* and *Sargassum*, transforming the benthic reef habitat to turf, with detrimental effects on native herbivores [98,99,100,101]. Nonindigenous *Caulerpa* species are invasive toxic algae that modify natural benthic communities, lowering the productivity of native macrophytes, displacing native species and changing the original structure of the macroalgal communities [102,103,104,105,106,107]. Therefore, the first step to be embarked upon after the current LEK activity should be to verify, where there is no certainty from our interviews, the occurrence of these species in the areas of interest and then to prepare mitigation management plans for the affected habitats. Some successful mitigation actions concern the progressive selective removal of invasive species by experts with the active participation of citizens, together with continuous monitoring and control of the affected sites [108]. In the case of *Caulerpa* spp., the removal activity should be promoted through the training of fishers and of other involved citizens, in order to avoid the further dispersal of the species’ vegetative parts (e.g., the thallus). Concerning rabbitfish, their selective removal should be managed by experienced staff, also involving local fishers, given the venomous nature of the species, and should be strictly controlled in MPAs. The removal will not manage to eradicate these highly invasive species but, if carried out systematically, will slow down their spread and allow for cost-effective population control. If no action is taken, in addition to a numerical increase in the population locally, the species in question are expected to expand westwards, as has already been the case for other Lessepsian species (e.g., *Portunus segnis*, *Fistularia commersonii*, *Lagocephalus sceleratus*, and *Parexocoetus mento*), either spontaneously or transported through maritime traffic [96,109,110,111].

The CIMPAL index calculated for each site allows identification of the highly impacted hotspot sites for non-native organism introductions, i.e., sites requiring priority action. This allows clearer identification of the site or sites where to prioritise the economic resources dedicated to management. In our case, the two sites in greatest need of environmental management—i.e., Fondali delle Isole Pelagieand Fondali dell’Isola di Capo Passero—are both affected by intense anthropic pressures (e.g., professional and sport fishing, maritime traffic and tourism at both sites, and agricultural activities at the latter site), with a consequent cumulative impact on the marine environments, suggesting the need for an ecosystem approach to restore the invaded habitats, rather than just an intervention on individual species. It is indeed known that there may be a relationship between the presence of invasive non-native species and the fragmentation of habitats, with the more fragmented habitats constituting the highest establishment opportunities for this type of species [112,113]. 

### 4.1. LEK Bias: Weaknesses and Strengths 

#### 4.1.1. Weaknesses

The increased involvement of citizens in scientific activities and the widespread use of social networks have recently introduced additional sources of information on alien species. However, data from these sources are not always verifiable, and their interpretation could lead to a distortion in the interpretation of biological phenomena. Photographic or video documentation is not always made available by the respondents, and misidentification of species can occur due to the sheer difficulty of identifying their distinctive morphological characters. This complicates the process of validation of records by researchers who must therefore rely on all their experience and common sense so as not to make errors. This implies a limited pool of target species that can be investigated, suggesting an exclusive focus on those species that are clearly identifiable by nonexperts. In some cases, however, uncertain information should also be considered, in particular when it deals with species hazardous to human health for which the attention threshold should be kept high. The involvement of the selected categories of citizens, i.e., different types of fishers and SCUBA divers, depending on the specific activity they perform and the area in which they operate, is fundamental. The more varied the sample of respondents, the greater the possibility of reporting species living in different environments (Figure 5), but it is not always possible to attain sufficiently high numbers of respondents for certain categories, both scuba divers and fishers. It thus becomes essential to use intermediaries, which facilitate access to those categories historically more reluctant to share information by mitigating any distrust they might harbour towards the interviewers.

#### 4.1.2. Strengths

The involvement of fishers and SCUBA divers in the LEK activity is fundamental, as both categories of citizens frequently work at sea and therefore represent the first sentinels of any environmental changes. The integration of these two categories represents a strong point of the study, since they explore different environments and with different methods; fishers capture organisms and can therefore provide concrete evidence of NIS occurrence from sea bottoms which are difficult to investigate directly, whilst SCUBA divers can obtain useful photographic material or videos in environments not explored by fishers. Even the categories of organisms retrieved by these two sea users are different: fishers have recognized experience on commercial species of fish, crustaceans, and molluscs, while divers can also provide information on algae and coastal organisms in general. Within the fishers’ category, a further choice concerns the type of fishery activity: trawl fishery operates at great depths and distance from the coast, contrary to small-scale and recreational fishery, all of them providing useful and complementary information according to their experience. The involvement of these citizen categories through the LEK activity also lays the foundations for the establishment of an early detection network, through which it will be easier to monitor certain ecological events so as to identify the most effective management strategies.

This LEK activity allowed the retrieval of data not available through standard monitoring or literature analysis, did not require excessive economic resources or time, and can be considered as an integrative approach to the study of NIS, helping to define the national baselines required by international directives. Indeed, this LEK activity even backdates a number of invasions, with respect to the literature records, as in the case of *A. dactylomela* and *E. anatina*.

The methods used simultaneously by the two countries proved to be easy to apply and could be replicated in other sites and in other countries by adapting them in the choice of habitats and species to be investigated. The use of shared protocols is in line with what is hoped for in the context of international directives both with regard to alien species and other environmental issues.

## 5. Conclusions

The information and data collected through the current LEK survey helped to discern the status of the studied areas and to retrieve information not available in the literature that would not otherwise have emerged. The LEK data were used to identify which N2K sites, within the Malta–Sicily region, are mostly affected by non-native species and to record and to rank the most impactful species. The study produced a valid scientific output in the form of site and species prioritisation in the definition of tools and strategies for future environmental management actions and mitigation measures.

The integration of the data collected from the Sicilian areas with those from the Maltese areas has made it possible to assess the strengths and weaknesses of the deployed method and then to create harmonised paths for the definition of common strategies for the protection of marine biodiversity in the cross-border area.

During the LEK survey, a network was also set up to retrieve documentation from the parties involved (photographs, videos, samples, etc.) regarding the presence of non-native species in the areas under study, providing new data, which integrated the information collected during the interviews [19].

## Figures and Tables

**Table 1:** N2K sites’ information investigated through LEK surveys in the Italian–Maltese area.

Country (MSFD Subregion)	Site Type	Site Code	Name	Marine Surface Area (ha)	1110 Presence	1120 * Presence	1150 * Presence	1170 Presence	8330 Presence
Italy (IT MWE)	pSCI (MPA)	ITA020047 (EUAP0555)	Fondali di Isola delle Femmine—Capo Gallo	2155	x	x		x	x
Italy (IT MWE, IT MIC)	SPA	ITA030042	Monti Peloritani, Dorsale Curcuraci, Antennamare e area marina dello Stretto di Messina	8117	x	x	x	x	
Italy (IT MIC)	SAC	ITA040012	Fondali di Capo San Marco—Sciacca	18,330	x	x		x	
Italy (IT MIC)	SAC (MPA)	ITA040014 (EUAP0553)	Fondali delle Isole Pelagie	4085	x	x		x	x
Italy (IT MIC)	SAC	ITA090028	Fondali dell’Isola di Capo Passero	5367	x	x		x	x
Malta (MT MIC)	SAC (MPA)	MT0000101	Żona fil-Baħar Bejn Rdum Majjiesa u Ras ir-Raheb	1459	x	x		x	x
Malta (MT MIC)	SAC (MPA)	MT0000102	Żona fil-Baħar fl-Inħawi ta’ Għar Lapsi u ta’ Filfla	2629		x		x	
Malta (MT MIC)	SAC (MPA)	MT0000103	Żona fil-Baħar fl-Inħawi tad-Dwejra (Għawdex)	229		x		x	x
Malta (MT MIC)	SAC (MPA)	MT0000104	Żona fil-Baħar fl-Inħawi ta’ Mġarr ix-Xini (Għawdex)	169	x	x		x	x
Malta (MT MIC)	SAC (MPA)	MT0000105	Żona fil-Baħar fil-Grigal ta’ Malta	15,880	x	x		x	x

x = presence of habitat; IT = Italy; MT = Malta; MWE = Western Mediterranean Sea; MIC = Ionian Sea and the Central Mediterranean Sea. MPA = Marine Protected Area; SAC = Special Area of Conservations; pSCI = proposed Site of Community Importance; SPA = Special Protection Area. 1110 = Sandbanks, which are slightly covered by sea water all the time; 1120 = *Posidonia* beds (Posidonion oceanicae); 1150 = Coastal lagoons; 1170 = Reefs; 8330 = Submerged or partially submerged sea caves. * Priority habitat type.

**Table 2:** Non-native species investigated through a LEK survey in the Italian and Maltese three MSFD subregions. The year of the first record is also reported with the corresponding reference. IT = Italy; MT = Malta; MWE = western Mediterranean; MIC = Ionian and Central Mediterranean. The dash indicate that the species has never been reported in the area.

	Species	IT MWE	IT MIC	MT MIC
Chlorophyta	*Caulerpa cylindracea* [37,38,39]	1993	1993	1999
	*Caulerpa taxifolia* [40,41,42]	1992	1993	2013
Tracheophyta	*Halophila stipulacea* [43,44,45]	1995	1988	1970
Ctenophora	*Mnemiopsis leidyi* [46]	2009	2009	–
Cnidaria	*Cassiopea andromeda* [47,48,49]	2014	2014	2009
Mollusca	*Aplysia dactylomela* [50,51,52]	2009	2002	2008
	*Bursatella leachii* [53,54,55]	1969	1968	1969
	*Melibe viridis* [56,57,58]	2007	1991	2008
	*Rapana venosa* [59,60]	1978	before 1988	–
Crustacea	*Callinectes sapidus* [61,62]	1964	1999	–
	*Percnon gibbesi* [63,64,65]	2000	1999	2001
	*Portunus segnis* [66,67,68]	2004	1966	1972
Pisces	*Abudefduf* spp. [69,70]	1957	–	2013
	*Acanthurus monroviae* [71]	–	–	2013
	*Cephalopholis* spp. [72,73]	–	2009	2008
	*Enchelycore anatina* [74,75]	–	2011	2013
	*Fistularia commersonii* [76,77,78]	2003	2002	2005
	*Parupeneus forsskali* [79]	–	–	maybe 1979
	*Plotosus lineatus*	–	–	–
	*Pterois miles* [80]	–	2016	–
	*Saurida lessepsianus* [81]	–	1978	–
	*Siganus luridus* [82,83]	2004	2003	1990
	*Siganus rivulatus* [84]	–	2015	–
	*Upeneus pori* [85]	–	2012	–

**Table 3:** Strategies for collecting good-quality LEK data.

Key Steps	Strategy
Citizen engagement	The respondents were recruited through trusted intermediaries, such as directors of fishing associations and MPA operators, after being informed of the ongoing activities carried out by researchers and the importance of their involvement in such activities. In particular, intermediaries were required to involve a diversified array of fishers in terms of the gear used, in order to obtain more exhaustive information.
Local knowledge of marine organisms and environment	Intermediaries were asked to involve mainly citizens with marine-related jobs (e.g., fishers, SCUBA divers), having a certain degree of marine experience.
Citizen skill and experience	The questions asked to the participating citizens were straightforward and suited to their competences. The skill level (beginner, basic, or advanced experience) was deducted from the declared experience in terms of years of activity at sea.
Identification and description of the species	In order to facilitate the species identification, a poster with 24 photos of selected non-native species was shown (Supplementary File), also specifying their distinctive characters from similar species.
Description of the observation	The respondents were invited to provide, whenever possible, supplementary information on the habitat, depth, distance from the coast, etc., of the site where the species was observed.
Location of the sighting	Detailed maps of the study area were shown, in order to properly locate the sightings.
Observation documentation	In order to validate the sightings, respondents were invited to provide any photographic/video material of the species sighted, as well as of other organisms they considered interesting to report.

**Table 4:** Impact weight for each non-native species per habitat type according to the classification proposed by Katsanevakis et al. [5]: Scale for the impact weights: ‘0’ minimal; ‘1’ minor; ‘2’ moderate; ‘3’ major; ‘4’ massive. The dash indicates that the species is not present in the habitat.

	Non-Native Species	1110	1120 *	1150 *	1170	8330
Chlorophyta	*Caulerpa cylindracea*	4	0	4	4	–
	*Caulerpa taxifolia*	4	0	–	4	–
Tracheophyta	*Halophila stipulacea*	2	0	2	–	–
Ctenophora	*Mnemiopsis leidyi*	–	–	2	–	–
Mollusca	*Aplysia dactylomela*	–	0	0	0	–
	*Bursatella leachii*	0	0	0	0	–
	*Melibe viridis*	0	–	0	–	–
	*Rapana venosa*	0	–	1	0	–
Crustacea	*Callinectes sapidus*	1	1	1	1	–
	*Percnon gibbesi*	–	–	–	0	–
	*Portunus segnis*	0	0	–	0	–
Pisces	*Abudefduf* spp.	–	0	–	0	–
	*Acanthurus monroviae*	–	–	0	0	–
	*Cephalopholis* spp.	0	–	–	0	0
	*Enchelycore anatina*	–	–	–	0	0
	*Fistularia commersonii*	0	0	–	0	–
	*Parupeneus forsskali*	1	–	–	1	–
	*Pterois miles*	–	–	–	4	–
	*Saurida lessepsianus*	0	–	–	–	–
	*Siganus luridus*	–	4	–	4	–
	*Siganus rivulatus*	–	4	–	4	–
	*Upeneus pori*	1	–	–	–	–

1110 = Sandbanks, which are slightly covered by sea water all the time; 1120 = *Posidonia* beds (Posidonion oceanicae); 1150 = Coastal lagoons; 1170 = Reefs; 8330 = Submerged or partially submerged sea caves. * Priority habitat type.

**Table 5:** List of non-native species and year of first sighting recorded during LEK activity in the three MSFD subregions; the columns “Already known” indicate records already reported in the literature as for Table 2; n.r. = not recorded during LEK activity. The columns “New” are the species records not previously reported in the literature; the dash indicates that the species record is not new in the MSFD subregions. MWE = western Mediterranean; MIC = Ionian and Central Mediterranean; IT = Italy; MT = Malta. The asterisk before the species indicates non-native species *sensu* MSFD; in brackets the number of records in the area; the symbol; ≤2019, corresponding to the year of the interview, was used when the year was not specified.

	Non-Native Species	IT MWE	IT MIC	MT MIC	IT MWE	IT MIC	MT MIC
		Already Known				New	
Chlorophyta	* Caulerpa cylindracea	2000 (4)	2008 (25)	2019 (1)	–	–	–
	* Caulerpa taxifolia	2009 (3)	2000 (18)	2019 (1)	–	–	–
Tracheophyta	* Halophila stipulacea	2009 (7)	2015 (9)	2019 (2)	–	–	–
Ctenophora	* Mnemiopsis leidyi	2012 (2)	2013 (4)	n.r.	–	–	2018 (2)
Mollusca	*Aplysia dactylomela*	**2000** (8)	**2000** (20)	2016 (8)	–	–	–
	*Bursatella leachii*	2015 (2)	2009 (3)	2018 (1)	–	–	–
	* Melibe viridis	n.r.	2017 (2)	n.r.	–	–	–
	* Rapana venosa	2015 (6)	≤ 2019 (3)	n.r.	–	–	≤2019 (1)
Crustacea	* Callinectes sapidus	n.r.	2008 (14)	n.r.	–	–	2019 (2)
	*Percnon gibbesi*	2008 (9)	2000 (28)	2004 (18)	–	–	–
	* Portunus segnis	2017 (1)	2009 (17)	2000 (6)	–	–	–
Pisces	*Abudefduf* spp.	≤2019 (2)	n.r.	n.r.	–	2016 (4)	–
	*Acanthurus monroviae*	n.r.	n.r.	2014 (1)	–	–	–
	*Cephalopholis* spp	n.r.	2013 (3)	n.r.	≤2019 (1)	–	–
	*Enchelycore anatina*	n.r.	**2000** (9)	2019 (1)	2018 (1)	–	–
	* Fistularia commersonii	2005 (15)	2003 (47)	2018 (4)	–	–	–
	*Parupeneus forsskali	n.r.	n.r.	2018 (6)	–	2017 (2)	–
	*Pterois miles	n.r.	2017 (2)	n.r.	–	–	–
	* Saurida lessepsianus	n.r.	1990 (4)	n.r.	≤2019 (2)	–	–
	* Siganus luridus	n.r.	2009 (28)	2000 (11)	–	–	–
	* Siganus rivulatus	n.r.	≤2019 (3)	n.r.	2014 (2)	–	–
	* Upeneus pori	n.r.	2017 (3)	n.r.	–	–	2018 (1)

In bold the species records previous to those reported in the literature as for Table 2. The underlined year corresponds to a registration validated by documentary material (photo/video).

## Data Availability

Data sharing not applicable.

## Supplementary Materials

The following supporting information can be downloaded at: https://www.mdpi.com/article/10.3390/biology12091158/s1, Supplementary File: Poster: Selected non-native organisms for LEK interviews.
