# Supplementary material for: Using Local Ecological Knowledge to Search for Non-Native Species in Natura 2000 Sites in the Central Mediterranean Sea: An Approach to Identify New Arrivals and Hotspot Areas
Source: Biology (Basel). 2023 Aug 23;12(9):1158. doi: 10.3390/biology12091158 (PMC10525112; doi:10.3390/biology12091158)
Supplement: Supplementary file 1 [file biology-12-01158-s001.zip › biology-2494082-supplementary.pdf]

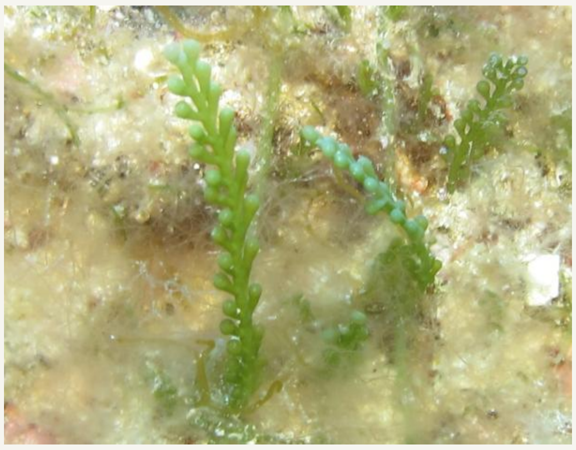

1 Caulerpa cylindracea
Photo: Pierpaolo Consoli

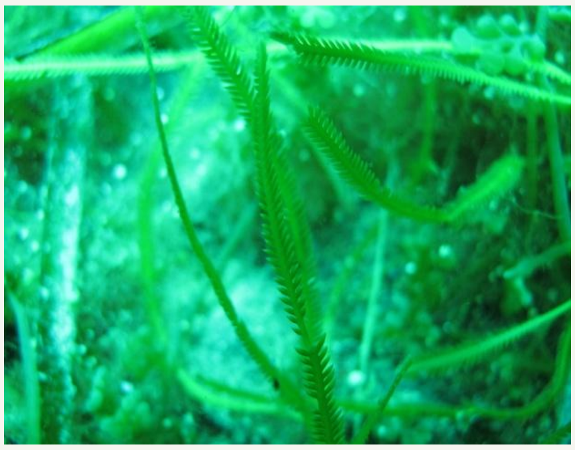

2 Caulerpa taxifolia
Photo: Alan Deidun

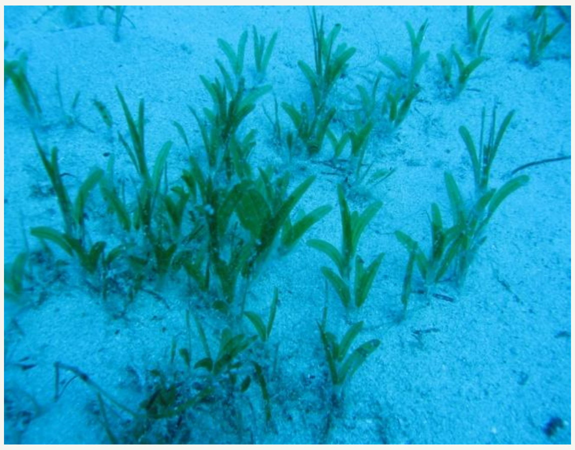

3 Halophila stipulacea
Photo: Alan Deidun

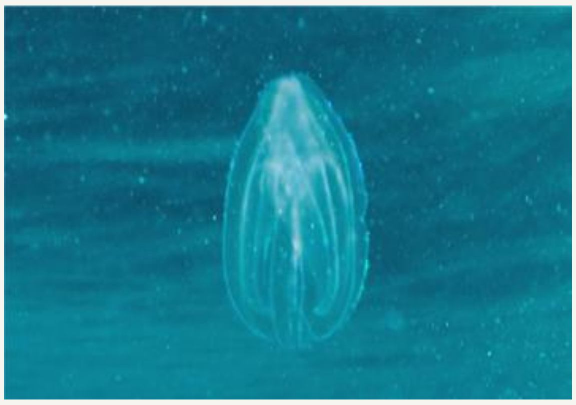

4 Mnemiopsis leidyi
Photo: Luca Gagliardi

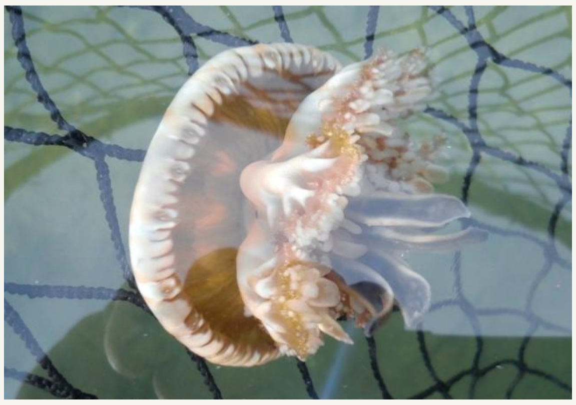

5 Cassiopea andromeda
Photo: Luca Castriota

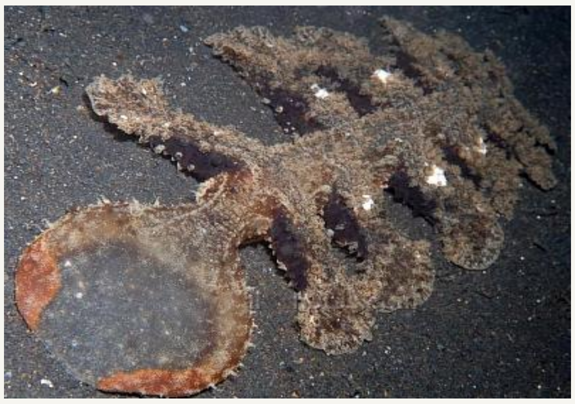

6 Melibe viridis
Photo: Mark Atwell

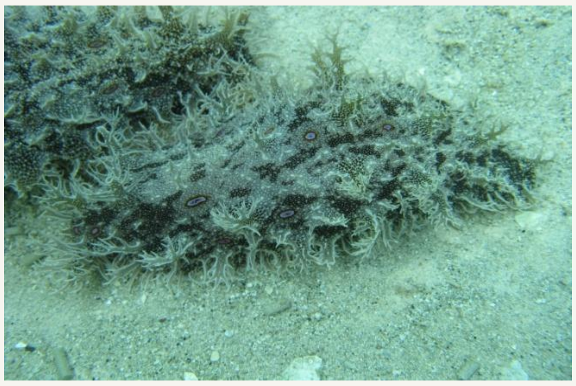

7 Bursatella leachii
Photo by: Alan Deidun

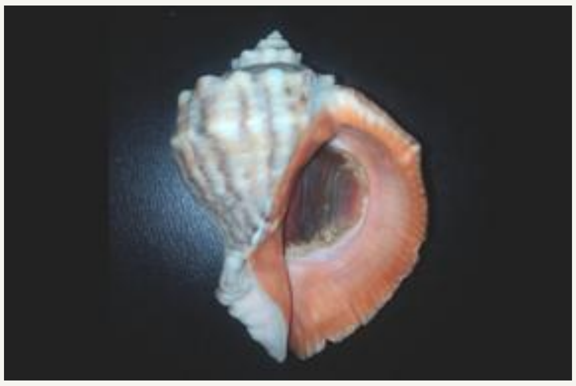

8 Rapana venosa
Photo by: Franco Agamennone

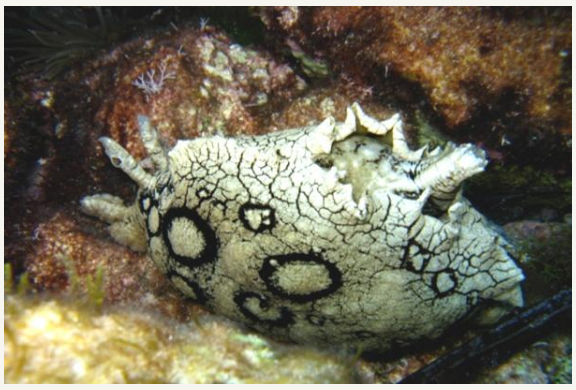

9 Aplysia dactylomela
Photo: Bruno Zava

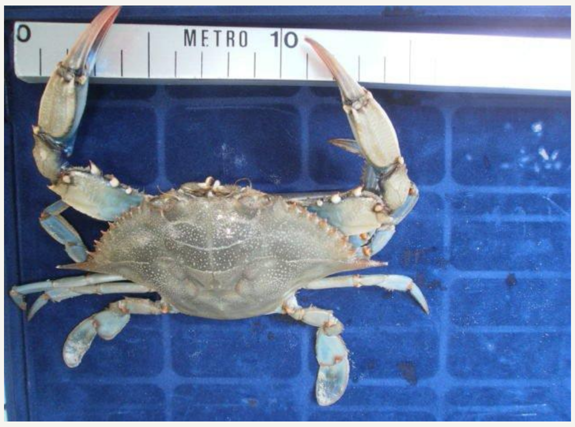

10 Callinectes sapidus
Photo: Roberto Costantini

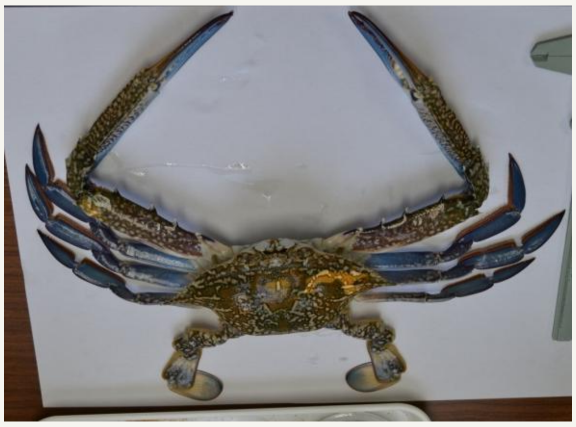

11 Portunus segnis
Photo: Alan Deidun

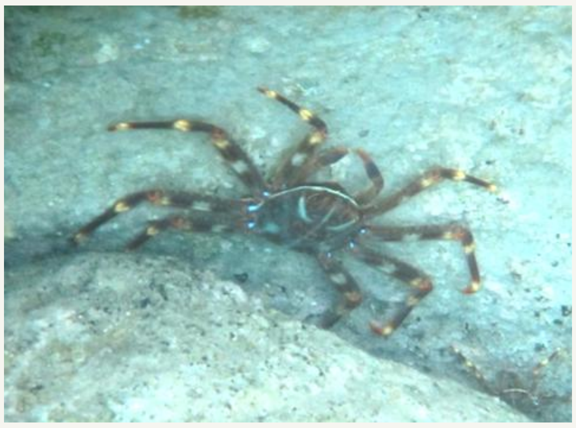

12 Percnon gibbesi
Photo: Alan Deidun

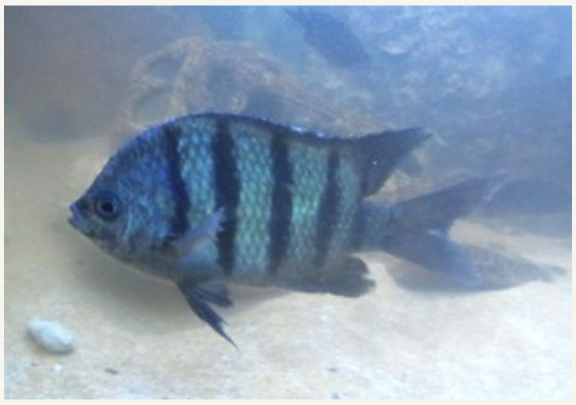

13 Abudefduf sp
Photo: Alan Deidun

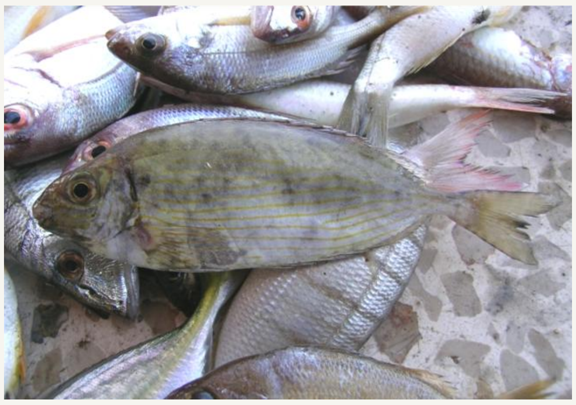

14 Siganus rivulatus
Photo: Pierpaolo Consoli

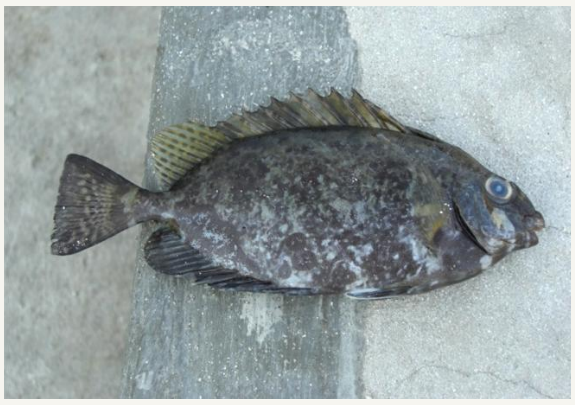

15 Siganus luridus
Photo: Alan Deidun

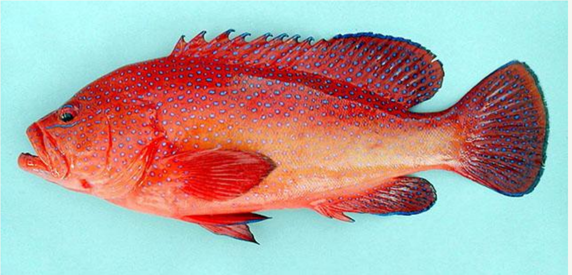

16 Cephalopholis taeniops
Photo: Pedro Cambraia Duarte

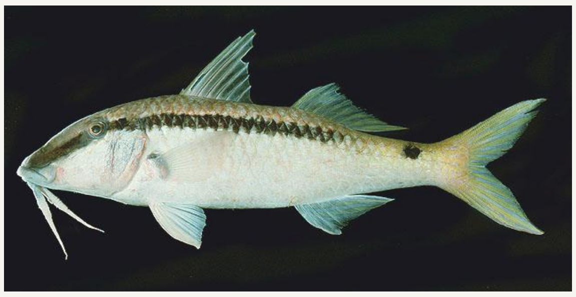

17 Parupeneus forsskali
Photo: John E. Randall

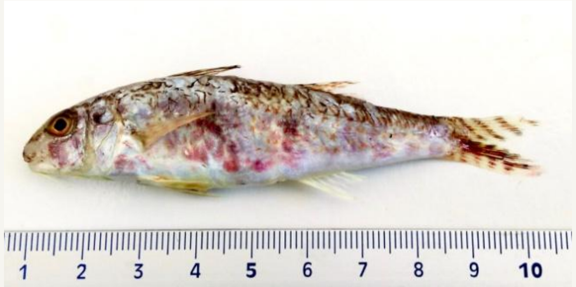

18 Upeneus pori
Photo: Bruno Zava

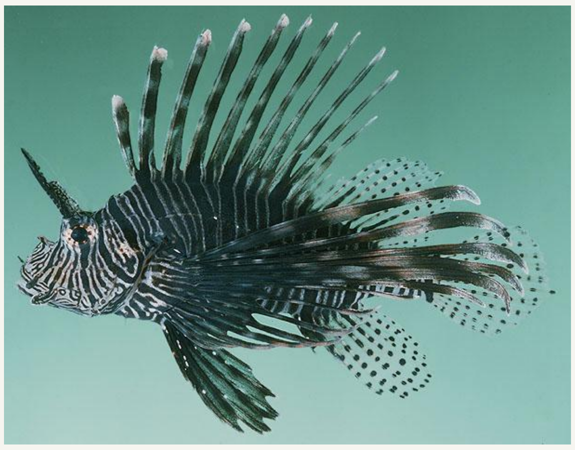

19 Pterois miles
Photo: John E. Randall

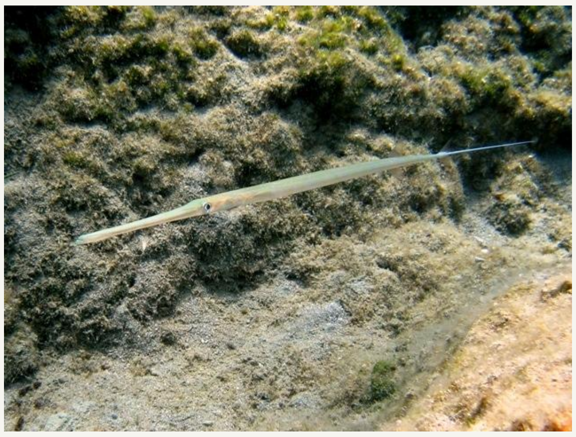

20 Fistularia commersonii
Photo: Roberto Pillon

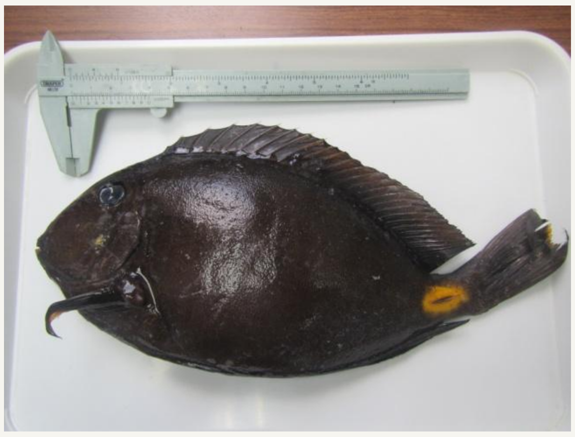

21 Acanthurus monroviae
Photo: Alan Deidun

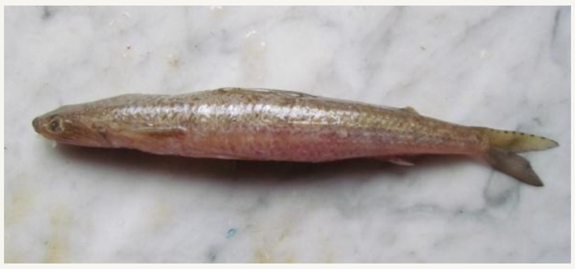

22 Saurida lessepsianus
Photo: Pierpaolo Consoli

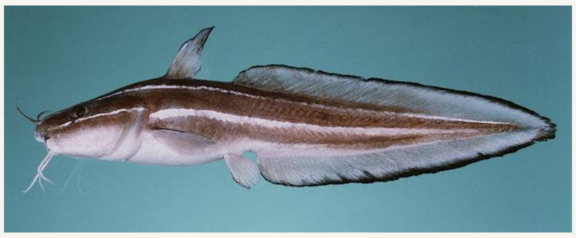

23 Plotosus lineatus
Photo: John E. Randall

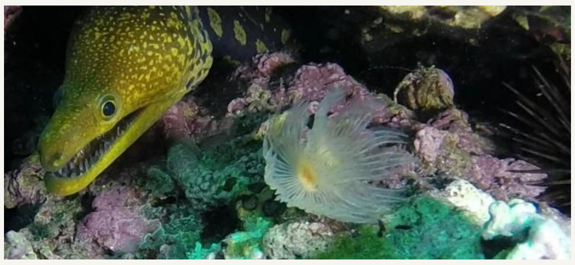

24 Enchelycore anatina
Photo: Giuseppe Mazza
